# Patient perceptions of care quality and discharge information following same‐day cardiac catheterization laboratory procedures: A mixed‐methods study

**DOI:** 10.1002/nop2.1578

**Published:** 2023-01-09

**Authors:** Kate Hames, Kevin White, Cherene Ockerby, Ruth Williams, Alison M. Hutchinson

**Affiliations:** ^1^ Monash Health Clayton Victoria Australia; ^2^ Monash Heart, Monash Health Clayton Victoria Australia; ^3^ Centre for Quality and Patient Safety Research – Monash Health Partnership, Monash Health Clayton Victoria Australia; ^4^ School of Nursing and Midwifery, Centre for Quality and Patient Safety Research Institute for Health Transformation, Deakin University Victoria Geelong Australia

**Keywords:** cardiac catheterization laboratory, discharge information, patient education, patient perceptions, same‐day procedure

## Abstract

**Aims:**

To examine patients' perceptions of care quality following a same‐day procedure in the cardiac catheterization laboratory and understand the extent to which they were prepared for discharge.

**Design:**

Single‐centre, mixed‐methods study.

**Methods:**

Postdischarge, online survey of patients who underwent a same‐day procedure in the cardiac catheterization laboratory (*n* = 150) and one‐on‐one interviews with 13 of these patients.

**Results:**

Survey responses were positive with mean scores between 4.39–4.83 out of five and 63.3% of respondents (*n* = 95) extremely likely to recommend the service to others. Interview data analysis identified three themes: the care experience, information and education for safe discharge, and follow‐up needs. Participants spoke highly of their interactions with clinicians and were satisfied with their care experience. Mode and content of information delivered varied, with some participants lacking guidance about postdischarge health management and clarity about follow‐up plans.

**Patient or public contribution:**

Participants were patients.

## 1 INTRODUCTION

To optimize safety and quality in health care, understanding patient experiences is important. Patients undergoing procedures in the cardiac catheterization laboratory (CCL), who are admitted and discharged on the same day, have limited time to ask questions or receive education about the procedure and postdischarge care. There is a paucity of research exploring patient perceptions of care quality in the CCL context, including communication, education and preparation for discharge. Therefore, the aims of this study were to explore patients' perceptions of care quality following a same‐day CCL procedure, and whether the discharge information and education provided prepared them for discharge following a same‐day CCL procedure.

## 2 BACKGROUND

Internationally, there has been rapid growth in CCL activity over the past two decades, with the number of percutaneous coronary interventions (PCI) observed in the United Kingdom increasing from 10,000 annually at the turn of the century to over 100,000 in 2013 (Lindsay et al., 2018), and over 1 million CCL procedures performed annually in the United States of America (Manda & Baradhi, 2018). In Australia, during 2014–15 more than 77,000 patients were admitted to hospital with cardiac conditions such as acute coronary syndrome (Saunders, 2018); data from 2013 suggested approximately 71% of Australian patients with myocardial infarction were treated in a CCL (Chew et al., 2013).

Cardiac catheterization laboratory offer a wide spectrum of procedures to investigate, diagnose and manage suspected or confirmed cardiac conditions such as coronary artery disease, cardiac arrythmias, valvular disease and heart failure (Manda & Baradhi, 2018). Whilst complications following same‐day CCL procedures are uncommon, estimated to occur in <1% of patients (Manda & Baradhi, 2018), patients must be supported in taking responsibility for their own postprocedural care after discharge, including self‐monitoring for potential complications (Bjørnnes et al., 2019; Kang et al., 2018).

In Australia, approximately 33% of all hospitalizations are for patients admitted and discharged on the same day in a public hospital (Australian Institute of Health Welfare, 2020). Same‐day procedures contribute to cost savings through decreased inpatient bed utilization for healthcare services (Liew et al., 2020), improved organizational efficiency, and lower costs for patients, contributing to patient satisfaction (Amin et al., 2018). Given the relatively short amount of time health professionals spend with patients following same‐day procedures, appropriate, comprehensive evidence‐based discharge planning is essential to ensure patients are informed about potential complications, symptom management, how to access support if needed, and resumption of usual activities (Gilmartin, 2007).

To support the delivery of evidence‐based care, a clinical care standard for patients with acute coronary syndrome was produced by the Australian Commission on Safety and Quality in Health Care (ACSQHC) (2019). One of the goals of the standard was to ensure patients receive the best treatment from the onset of symptoms, through to discharge from hospital (ACSQHC, 2019). Whilst the standard endorses the use of an individualized care plan to guide care after leaving the hospital, the focus of the care plan is on lifestyle changes, medications, psychosocial needs and referrals to specialists and cardiac rehabilitation (ACSQHC, 2019). The standard does not address other more immediate needs such as discharge information and education related to immediate and ongoing self‐care following a same‐day procedure.

There is a paucity of research that examines the patient experience and perceptions of care quality following same‐day procedures in the CCL. Furthermore, little is known about whether patients feel adequately educated or prepared for postprocedural self‐care following a same‐day CCL procedure, and how these factors contribute to patient perceptions of care quality. Therefore, the question guiding this research was “what are patients' perceptions of care quality and discharge information and education following a same‐day procedure in the CCL?”

## 3 METHODS

### 3.1 Design

This study used a concurrent mixed‐methods approach, comprising a survey and interviews with patients, based on naturalistic inquiry (Lambert & Lambert, 2012). By combining rich, subjective insights from individual interviews with more generalizable, standardized data obtained from surveys (Regnault et al., 2018), the mixed methods approach provided a more comprehensive understanding of the patient experience and perceived care quality (Zarei, 2015). Reporting followed the Strengthening the Reporting of Observational Studies in Epidemiology checklist for cross‐sectional studies (Appendix S1) and the Standards for Reporting Qualitative Research checklist (Appendix S2).

### 3.2 Setting

This study was conducted at a CCL at a major metropolitan public hospital in Melbourne, Australia. This site is a leading cardiac facility, with a 24‐h CCL service providing a wide spectrum of diagnostic and interventional coronary and structural cardiac procedures for patients. The facility performs in excess of 1400 PCI procedures and 3300 coronary angiograms per year.

### 3.3 Sample

Convenience sampling was used to identify patients who attended same‐day procedures in the CCL. Convenience sampling occurs when people are invited to participate in the research because they are convenient to access and/or readily available (Schneider et al., 2016).

#### 3.3.1 Recruitment procedure

As part of the existing process in the CCL, on the day following discharge, patients were contacted using an automated email sent via the electronic medical record system with an invitation to complete an online survey. There was no predetermined sample size for the survey, and all surveys completed during the data collection period were included.

Interview participants were recruited by independent clinicians in the CCL who were not part of the research team. These clinicians ensured patients met the inclusion criteria (i.e., able to speak and understand English, aged 18 years or more, feeling well enough to participate, and undergoing same‐day procedure) and provided eligible patients a brief explanation of the research. Patients who were willing to participate received the Participant Information Form and provided their contact details. A researcher contacted the patient approximately a week following discharge, providing time for patients to recover from their procedure whilst still being able to recall details about their experience. The researcher provided a verbal overview of the study, and potential participants were given an opportunity to ask questions, before confirming their consent to participate. The final number of participants was based on continuous evaluation of whether or not there was adequate information power to answer the research question (Malterud et al., 2016). Information power uses multiple factors including the aim, specificity, theory, dialogue and analysis of the data to ensure adequate sample size is determined (Malterud et al., 2016). Based on past experience and literature (Schneider et al., 2016; Trotter II, 2012), we expected this to be reached between 12–20 interviews.

### 3.4 Data collection

Survey data were collected for 12 months commencing 5 May 2021 via an automated online survey. A survey was already in use in the CCL for the purposes of monitoring care quality and consumer experience. The survey was derived from the Australian Hospital Patient Experience Question Set (AHPEQS) (Jones et al., 2021) and adapted for use in the health service by a committee of multidisciplinary healthcare professionals and consumers. The survey included 16 items about patients' confidence and trust in clinicians, the cleanliness of facilities, provision of information, education and discharge preparation. The researchers reviewed the survey and made modifications to improve readability (e.g., remove double‐barrelled questions) and standardize the response format to a single five‐point Likert scale ranging from Strongly Agree to Strongly Disagree, with a neutral mid‐point, for all items. Cronbach's alpha for the scale was 0.93 in this study. In addition, the survey captured participants' age, sex and CCL procedure. Since the survey was already in place in the organization, it was not possible to pilot test it prior to use. Permission was sought from the organization to access the data for the purpose of this research.

Individual interviews were conducted in July and August 2021 via telephone or Zoom videoconferencing, due to social distancing and other public health measures associated with the Sars‐CoV‐2 (COVID‐19) global pandemic. An interview guide was developed by the research team to provide a more in‐depth understanding of the questions asked in the survey. The interview guide comprised open‐ended questions designed to elicit participants' perceptions of their experiences in the CCL, and their preparation for discharge and recovering at home (Appendix S3). The interview guide was not piloted but its semi‐structured design allowed the interviewer flexibility to adapt questions based on participant responses. Interviews were conducted by a registered nurse who was undertaking a research‐based postgraduate qualification whilst working in the clinical setting. The members of the research team met regularly throughout the data collection phase to discuss the conduct of the interviews and reflect on the process. Interviews lasted 6–25 min (average 10 min) and were recorded via the Zoom application (including interviews conducted via telephone). The recordings were transcribed and labelled with unique identifiers, to ensure participants could not be identified (National Health and Medical Research Council, 2018). Participants were offered an opportunity to review their interview transcript to make additions or corrections but no participants accepted this offer.

### 3.5 Data analysis

Survey data were analysed descriptively using IBM SPSS Statistics v26 (IBM Corp., 2019), including frequencies and mean scores for scale items. Percentages were adjusted to accommodate a small number of missing responses for some items. Missing responses were excluded pairwise. The first author led the analysis of the interview data using Braun and Clarke's (2012) six phases of thematic analysis, with regular consultation and guidance from two members of the research team. This process was undertaken using Microsoft Excel (Microsoft Corporation, 2021).

### 3.6 Ethical considerations

The study was approved by the human research ethics committee at the health service (Ref: RES‐20‐0000812L‐67955) and the university (Ref: 2021‐153). Participation was voluntary. Survey consent was implied by submission of the survey. All potential interview participants were informed about the study by independent clinicians who were not involved in the research. All interview participants received detailed participant information, had an opportunity to ask questions and provided informed consent prior to the commencement of their interview. Survey responses were anonymous and interview transcripts were de‐identified to maintain anonymity, with data stored on secure, password‐protected servers. Anonymity and confidentiality were further protected by the reporting of findings in aggregate form.

## 4 RESULTS

### 4.1 Survey findings

Table 1 provides an overview of the key personal and clinical characteristics of respondents (*n* = 150). Respondents' ages ranged from 19–96 years (Mdn = 67, IQR = 18).

**TABLE 1:** Personal and clinical characteristics of respondents (*n* = 150)

Variable	Response	*n*	%
Gender	Female	47	31.3
	Male	103	68.7
Highest education	Primary school	4	2.7
	Secondary school	61	40.7
	TAFE/trade	37	24.7
	University/college	48	32.0
Living arrangements	Live at home with family	118	78.7
	Live alone	27	18.0
	Live in residential care	1	0.7
	No response	4	2.7
Procedure type	Angiogram	104	69.3
	Electrophysiology study/ablation	21	14.0
	Pacemaker insertion/generator change	20	13.3
	Loop monitor insertion	4	2.7
	Automatic internal cardiac defibrillator insertion	1	0.7
Care location	CCL immediate recovery area	117	78.0
	Cardiology day procedure unit	33	22.0

Responses to survey items are outlined in Table 2. Overall, respondents reported very positive perceptions of their experience in the CCL. Mean scores ranged from 4.39–4.83 out of five (Table 2). With regard to discharge preparation, the majority of respondents “Strongly Agreed” that, before they left hospital, the nurses and the doctors gave them sufficient information about managing their health and care at home, and that their family or home situation was considered when planning for discharge (Table 2).

**TABLE 2:** Responses to individual survey items (*n* = 150)

Items	*N*	M	SD	Disagree		Neither disagree or agree		Agree		Strongly agree	
				*n*	%	*n*	%	*n*	%	*n*	%
I had confidence in the staff treating me.	149	4.81	0.41	0	0	1	0.7	26	17.4	122	81.9
I had trust in the staff treating me.	150	4.83	0.39	0	0	1	0.7	23	15.3	126	84.0
The staff explained things in a way that I could understand.	150	4.68	0.58	2	1.3	3	2.0	36	24.0	109	72.7
I was given an adequate amount of information about the procedure I consented to.	150	4.66	0.60	2	1.3	4	2.7	37	24.7	107	71.3
I was given an adequate time to ask questions about my procedure.	149	4.64	0.61	3	2.0	1	0.7	43	28.9	102	68.5
I felt I was appropriately included in decisions made about my care and treatment.	149	4.60	0.54	0	0	4	2.7	52	34.9	93	62.4
If there were delays to receiving care, I was provided with updated information on how long I would have to wait in the waiting area.	147	4.39	0.74	3	2.0	14	9.5	53	36.1	77	52.4
If I needed assistance, I was able to get help within a reasonable time.	150	4.61	0.55	0	0	5	3.3	48	32.0	97	64.7
The rooms that I was cared for in were clean.	150	4.69	0.52	0	0	4	2.7	38	25.3	108	72.0
The toilets and bathrooms that I used whilst in hospital were clean.	134^a^	4.54	0.64	2	1.5	5	3.7	46	34.3	81	60.4
Before I left hospital, the doctors gave me sufficient information about managing my health and care at home.	149	4.48	0.83	6	4.0	15	10.1	30	20.1	98	65.8
Before I left hospital, the nurses gave me sufficient information about managing my health and care at home.	148	4.68	0.60	2	1.4	4	2.7	34	23.0	108	73.0
The hospital staff took my family or home situation into account when planning my discharge.	149	4.59	0.63	2	1.3	5	3.4	45	30.2	97	65.1
When I left hospital, I was confident that all of my care needs had been organized.	146	4.48	0.72	3	2.1	10	6.8	47	32.2	86	58.9
I felt that I was given adequate time to recover before being sent home.	149	4.65	0.54	0	0	5	3.4	45	28.2	102	68.5
Overall, I was satisfied with the care I received.	150	4.71	0.51	0	0	4	2.7	36	24.0	110	73.3

*Note*: No respondents selected “Strongly disagree” for any items so this response option is omitted from the table.

^a^ 14 respondents indicated this item was Not Applicable; a further 2 responses were missing.

The final item on the survey asked how likely respondents would be to recommend the CCL to a friend or colleague. The mean score for this item was 9.36 (SD = 1.37), with the majority of respondents (*n* = 95, 63.3%) selecting the maximum rating of 10 (extremely likely).

### 4.2 Interview findings

Thirteen patients participated in an interview, including eight males and five females. Eleven participants underwent an angiogram, of whom, three also underwent PCI. Of the remaining two participants, one underwent an Automatic Implantable Cardiac Defibrillator insertion and the other a Supraventricular Tachycardia ablation. Eleven patients were discharged from the cardiac day procedure ward at the facility, one was discharged directly from the CCL, and another was unsure of their discharge location.

#### 4.2.1 Themes identified

Three main themes were identified: (1) the care experience, (2) information and education for safe discharge and (3) follow‐up needs.

*(1) The care experience*

Participants reflected on their interactions with clinicians and their overall experience. This theme comprises three sub‐themes: (i) interactions with clinicians, (ii) negative care experiences and (iii) positive care experiences.

(i) Interactions with clinicians

This sub‐theme concerns participants' reflections on communication and interactions with clinicians (including doctors and nurses). Most participants spoke highly of clinicians, stating:

The clinicians were fantastic, totally professional. (Participant 13)

Look, I was extremely happy because whoever I dealt with were polite and they were terrific. I've got no complaints at all. (Participant 11)

There's not one bad person in that room… [they] wheeled me to the car actually. So you know, you can't ask for a better than that. (Participant 3)

However, one participant described a negative experience of communication from one doctor:

I was supposed to have a female doctor and then it turned out to be a male surgeon and he goes, “if an accident happens, you'll need a pacemaker” and I'm like ‘accident?’, I'm like “mmm”, so his wording wasn't overly wonderful. (Participant 5)

The same participant also felt the nurses did not appropriately explain what was happening:

Every nurse should know that you explain the procedure…So they didn't even tell me what they were putting in at the time, you know the bedside manner was just shocking. (Participant 5)

This contrasted with the experience of other participants who described feeling well informed:

They informed me all the way through the thing…their bedside manner is good, it is very good and if you ask a question, they answer, and you are totally put at ease. (Participant 9)

Everyone explained things prior to receiving them…As far as communicating what was going on at the time, it was really good…It was really easy to understand. (Participant 7)

(ii) Negative care experiences

Negative care experiences for participants related to physical pain, or the fear of pain, a lack of patient‐centred care and lack of clinician support during their hospital stay.

The pain was atrocious… it was horrible I would not do it again, I'd rather die… (a) I felt alone and (b) I don't think the pain was well managed, like you know, a light sedation for what I went through. Ugh… you know three nights I cried thinking that pain would come back. (Participant 5)

This participant also explained they tried to ask for help but felt nobody was there:

I was on the other side of whatever their table was yeah. I'm going “hello is anyone there” you know, no one was around me yeah. And I was just in that much pain… (Participant 5)

Conversely, another participant was concerned about pain but understood it was likely a short‐term impact from the procedure:

I had a few little niggly pains and I thought to myself, don't panic, cause I knew they were up in that area and you know, you do get a bit scared… (Participant 11)

Another participant expressed anger as expectations for their care were not met. The participant reported being previously informed about blockages in their arteries that needed to be resolved, but was then unable to get them resolved on the day of the procedure:

That probably made me a little bit angry, because the fact was, I was there on the day to get something done and I didn't think it was an issue and then all [of a] sudden became I didn't get it done. (Participant 3)

This same participant then described feeling disappointed as the doctor explained to them that he would need to consult his colleague to be able to fix the blockage:

…he said to me that it's out of his league, he'll have to get one of his partners to do that one yeah. I understand that. But that to me sounded quite serious but nobody seems to be taking it seriously. (Participant 3)

Finally, another participant described feeling rushed by the nursing staff to leave the ward postprocedure:

I was just a little bit disappointed, and I was the only person on the ward, so I felt as though I was being rushed out so they could close the ward up… I was sort of a little taken aback. (Participant 6)

(iii) Positive care experiences

Participants were asked to describe how they felt when discharged from hospital. Many participants spoke positively about their CCL procedure and the care they received.

…so I don't think they could do it any better than they did. (Participant 11)

I was very pleased with it…the whole experience was very good. (Participant 6)

One participant also spoke highly about the preparation they received prior to coming to hospital:

[The preparation was] very good, the pre‐preparation like contacting me going through everything and then the paperwork was quite a breeze actually. (Participant 3)

*(2) Information and education for safe discharge*

Three sub‐themes were aligned with this theme: (i) varying modes and content of information at discharge, (ii) understandability of discharge information and education and (iii) preparedness for postdischarge self‐care.

(i) Varying modes and content of information at discharge.

Participants described considerable variation in the scope, detail and mode of delivery of discharge information provided following their same‐day procedure, with some not recalling any discharge information being provided. Some participants described receiving written information:

Yeah, there was a handout. If you think there's any discharge or bleeding yeah go to emergency. (Participant 4)

I was given an instruction sheet to sort of say, don't do this, don't do that sort of thing. (Participant 6)

Two participants explained they were given verbal instructions only:

I didn't get any paperwork to tell me, but I got told by the girl that took me out to the door and she said if that starts bleeding she said just ring an ambulance. (Participant 3)

What they did tell me, if I had any bleeding, to call an ambulance. (Participant 2)

Others recalled receiving both written and verbal discharge instructions:

…I was given instructions to read… And I was told what to do, and what not to lift and to rest (Participant 9)

She gave me a sheet of paper which she more or less repeated, which said don't lift anything for a week and if it spurted out, put pressure on it and yes. So I knew what to do (Participant 11)

Some participants recalled receiving instructions about what to do if bleeding was to occur when they were discharged home. However, others could not recall whether they received any instructions.

I cannot remember that they did [give instructions]. (Participant 8)

I can't remember getting any [instructions] but that doesn't mean I didn't get it. (Participant 10)

(ii) Understandability of discharge information and education

In relation to understanding and recalling discharge instructions, responses varied from feeling the instructions were clearly delivered and easy to understand to a perception that there was too much information provided, often overwhelming patients. Specifically, some participants felt the repetition of information (being both written and verbal) was unnecessary:

There was a handout but obviously by then I had been told twice…Oh definitely, on more than one occasion. The nurses… gave me the background and then the surgeon [doctor] came in and did the same thing just this is what you've got to look out for. Anything like this happens, just call 000, just get back here. (Participant 7)

In response to whether instructions were clear, a participant stated:

I felt that what they said I understood. (Participant 5)

Some participants reported feeling disorientated and overwhelmed by their experiences, which affected their memory of receiving, recalling and/or understanding information and education. Consequently, one participant highlighted the importance of being able to take written information home, to read in their own time, arguing that patients are not in a condition to adequately comprehend information and education whilst recovering from sedation.

Um to be very honest with you, I think I was a bit overwhelmed as well, so I can't, I don't think I remember everything…I do remember bits of it, but not everything, so I guess that was me being maybe 100 things going in my head. (Participant 1)

…but mind you you're under whatever you're under… I struggle listening to stuff fully anyway…so for that not to be printed out and I would have brought it home and had a read, but I didn't get anything like that. (Participant 3)

(iii) Preparedness for postdischarge self‐care

Not all participants felt they had a clear understanding of the activities they could resume or the status of their health beyond their procedure. One participant reported an issue with their arm swelling postprocedure. Given this experience, they stated they needed more information about what to expect or what to do if a complication was to occur:

But I've also had complications with my arm since the angiogram that sort of, I presume is the normal thing. But because I wasn't advised of anything, I don't know? … I would have liked a little bit more information [about] what to expect. (Participant 6)

Two participants reported not having a clear understanding of their health status beyond discharge:

I don't know what I can and can't do heart wise. Do I exercise, do I do this, do I… I mean I had seven days off work yeah that's fine yeah and monitor the groin for infection…but you know, like no one told me what I can resume doing…And I asked the ward nurse, and she wasn't sure. I was like I can't be bothered. (Participant 5)

I'm still wondering what the…hell I got. Walking around with a little bit of chest pain now and again out of breath a lot. Yeah, no I don't know what's going on. (Participant 3)

*(3) Follow‐up needs*

The theme “Follow‐up needs” illustrates contrasting views of participants, with some feeling well informed and others not knowing their follow‐up plan postdischarge. Two participants were instructed to see their general practitioner (GP) after discharge:

I did, I've just been with my GP today. (Participant 9)

They just said to go back to my own doctor in a week to check the wound. (Participant 11)

Whilst one participant knew to follow up with their GP, they were unsure of plans to follow up with the cardiologist:

I made an appointment with my local doctor… I'm already down for a cardiologist so I probably would have told them that. I can't remember talking about a cardiologist with anyone. (Participant 10)

Most participants stated they were unsure about follow‐up plans beyond their procedure with many reporting they did not receive answers or know what to do beyond discharge.

I didn't think I got any answers that day. The only thing… paperwork I walked out with was the… [doctor] has organised me to have the stress test and that was all I really knew. Yeah I just thought I still don't know anything. (Participant 3)

When one participant was asked if they were still waiting to hear from the doctors about a plan, they confirmed that that was the case. Another participant asserted the follow‐up process was difficult to comprehend:

If I was on my own at home and being let out and organise your own cardiologist and maybe I am not as in tune as what other people are. I would find that difficult to process. (Participant 7)

This participant consequently warned that patients may miss future appointments due to unclear processes for follow‐up:

It's quite hard to absorb everything so some people might just get missed because they don't absorb that they've got to organise it and maybe they'll sit at home and wait because they haven't or can't or don't read the information they're given. (Participant 7)

## 5 DISCUSSION

This study sought to explore patients' perceptions of care quality and discharge information and education following a same‐day procedure in the CCL. The survey findings indicated that patients had a very positive experience and would be extremely likely to recommend the service to others. Similarly, most interview participants felt that the clinical/procedural care, as well as interactions and communication with clinicians, contributed positively to their overall experience. Nonetheless, the interviews afforded patients an opportunity to provide more in‐depth feedback about their experience and identified areas for improvement, particularly in relation to preparation for discharge.

Patient‐centred communication adapted to individual needs has previously been shown to contribute to perceptions of overall satisfaction with the care experience (Esmaeili et al., 2016; Franzon et al., 2018; Harrison et al., 2017). Similar to Harrison et al. (2017), the findings from this study suggest that patients' positive perceptions of their care experience were influenced by whether they felt well‐informed about their procedure. Almost all survey respondents agreed that staff explained things in a way they could understand, and they were given adequate time to ask questions about their procedure. This finding was reiterated in the interviews, with participants speaking highly about how clinicians communicated with them before and during their admission, taking the time to provide clear and thorough explanations, and answer questions in an accessible manner. Establishment of a caring relationship between nurse and patient is integral to patients feeling emotionally and physically safe, valued and respected (Harrison et al., 2017; Wiechula et al., 2016). Hence, ensuring care quality through the establishment of a caring relationship is essential to patient perceptions of, and satisfaction with, care (Fatima et al., 2018). Of note, the survey item with the lowest mean score asked about being kept informed in the event of delays, so this may offer an opportunity to improve communication with patients in the CCL.

Comprehensive patient education prior to discharge, including information about diagnosis, treatment and self‐care, is key to facilitating a successful transition from hospital to home and has been found to reduce readmission rates for patients who have undergone cardiac catheterization (Yan et al., 2011). Survey respondents in this study typically agreed that both the doctors and nurses gave them sufficient information about how to manage their health and care at home before they left hospital. The interviews provided an opportunity to explore this in more detail and the findings indicated variation in how education and information were provided prior to discharge. Whilst some participants reported receiving written discharge instructions, others reported they only received verbal instructions, and some described not receiving any instructions. These findings suggest the process was inconsistent and may not have been effective in supporting patients to self‐care following discharge.

Previous research has identified that the provision of information to patients in written form only, such as a discharge instruction sheet, may lead to sub‐optimal outcomes because patients do not typically have the appropriate skills or knowledge to effectively self‐care (Horwitz, 2017). In particular, Franzon et al. (2018) identified lower levels of satisfaction with written discharge instructions for those aged ≥80 years, females, and those with higher levels of health literacy. Provision of both verbal and written discharge information may be beneficial in communicating key information and minimizing misunderstanding (Theodoridis et al., 2020), standardizing the processes for discharge education (Kang et al., 2020), and better preparing patients for discharge. Tailoring the content of the discharge education to patient characteristics and learning needs and preferences can significantly increase patient satisfaction (Franzon et al., 2018) and enhance patients' overall knowledge and ability to self‐care safely after discharge (Kang et al., 2018). Moreover, a systematic process for discharge preparation can lead to more positive perceptions of care quality and lower readmission rates (Yan et al., 2011). Our interview findings suggest that a clear, consistent and systematic process for providing education and discharge information for patients in the CCL, in verbal and written form, is warranted and may improve patients' ability to, and confidence with, self‐care and their overall experience.

Just as the content of discharge information is important, so too is the timing, a factor that may impact patients' ability to recall the information (Theodoridis et al., 2020), particularly if they have received sedative medication. For example, discharge education provided to patients following same‐day pacemaker insertion under conscious sedation found education given within 1 h was much less effective than education given after 3 h (Schuster et al., 1999). The short timeframe for discharge information and education delivery with increased uptake of same‐day procedures presents a challenge in maintaining patient safety beyond discharge (Schuster et al., 1999).

When patients are not given adequate discharge information, their recovery and ability to self‐care can be negatively impacted (Bartoletti et al., 2019; Kang et al., 2018). Ideally, the patient should be placed at the centre of the process, with shared decision‐making between the patient, health professional and, when needed, the family and/or carer (Rushton et al., 2017). Given that variability in patients' understanding and recall of information and discharge, education was identified in the interview findings, involving family and/or carers in discharge education may be helpful to ensure patients are able to be discharged safely. This study was conducted during the COVID‐19 pandemic and restrictions on visitors to hospitals may have decreased family and carer participation in health management and the discharge processes. Nonetheless, the majority of survey respondents perceived that the hospital staff took their family or home situation into account when planning their discharge.

The benefits of same‐day procedures such as decreased length of stay, reduced rates of nosocomial infections, decreased healthcare expenditure and increased patient satisfaction have been previously well researched (Bertrand et al., 2007; Chen et al., 2019; Kim et al., 2013). However, this study suggests information provided to patients about care progression beyond their same‐day procedure should be made clearer to ensure patients understand their follow‐up plan. Whilst it was beyond the scope of this study to measure the duration of patient admissions (e.g., in hours), it is possible that patient discharge was expedited to minimize risk in the context of the COVID‐19, limiting the time available for clinicians to provide discharge education and information.

Patients typically have unique ongoing needs postdischarge, which may require follow‐up with a primary healthcare provider such as their general practitioner or cardiologist. Some patients consider their CCL procedure is a complete “fix” for their health problems (Young & Barnason, 2015); however, it is likely that a proportion of patients will require ongoing follow‐up and health management (World Health Organization, 2021). Despite a lack of research regarding follow‐up post same‐day CCL procedures, establishing a clear follow‐up plan for patients before they are discharged from hospital is important to ensuring ongoing health management and patient safety.

## 6 LIMITATIONS

The findings of this single‐site study reflect local practices associated with the CCL and the cardiac ward at the respective health service, and therefore may not be transferrable to other settings. The exclusive use of an online survey, with no option for completion of a paper survey, is acknowledged as a source of potential bias. Furthermore, the survey was already in use at the health service and, as such, it was not possible to pilot test the survey prior to implementation. Public health and infection control measures imposed by the health service in response to the COVID‐19 pandemic limited the researchers' ability to visit the CCL to promote the study and encourage clinicians in the CCL to assist with patient recruitment for interviews. For pragmatic reasons, a convenience sampling approach was therefore adopted and it is unclear how well the perceptions of the participants represent the overall CCL patient population. We note, however, that the samples for both the survey and interviews were representative of the general Australian cardiac patient population in terms of the higher proportion of men (Chew et al., 2013; Lefkovits et al., 2022) and angiography being the most common procedure (Chew et al., 2013).

## 7 CONCLUSIONS

This study was designed to elicit patients' perceptions of care quality and discharge information and education following a same‐day procedure in the CCL. Overall, both survey and interview findings suggested patients' perceptions of the quality of care were positive. A strength of this study, however, was that interviews provided an opportunity to explore the patient experience in more depth. In particular, the findings identified that preparation for discharge varied, with some participants feeling well prepared for discharge, whilst others felt inadequately prepared and lacked vital information to facilitate post‐discharge self‐care. The varied responses suggest patients' care needs require more detailed assessment to ensure they are aware of, and understand, the discharge education provided to them. Furthermore, follow‐up plans should be made clear to patients attending a same‐day procedure to ensure their care continues beyond their discharge and the immediate recovery phase.

## AUTHOR CONTRIBUTIONS

All authors have made substantial contributions to conception and design, or acquisition of data, or analysis and interpretation of data; they have been involved in drafting the manuscript or revising it critically for important intellectual content; they have given final approval of the version to be published; and agreed to be accountable for all aspects of the manuscript.

## FUNDING INFORMATION

No funding was received for this research.

## CONFLICT OF INTEREST

The authors have no conflicts of interest to declare.

## ETHICAL APPROVAL

The study was approved by the human research ethics committee at the health service (Ref: RES‐20‐0000812L‐67955) and Deakin University (Ref: 2021‐153).

## Supporting information


Appendix S1.

Appendix S2.

Appendix S3.

## Data Availability

The data that support the findings of this study are available from the corresponding author upon reasonable request.
