# Supplementary material for: Patient perceptions of care quality and discharge information following same‐day cardiac catheterization laboratory procedures: A mixed‐methods study
Source: Nurs Open. 2023 Jan 9;10(5):3263–73. doi: 10.1002/nop2.1578 (PMC10077407; doi:10.1002/nop2.1578)
Supplement: Supplementary file 3 — Appendix S3. [file NOP2-10-3263-s003.docx]

**Interview guide**

**Pre-recording**

Thank you so much for agreeing to participate in an interview.

I just wish to confirm that you have read the Participant Information Sheet and you are willing to consent to participate in this interview?

I want to remind you that you can stop the interview at any time and if you don’t wish to answer a particular question, you don’t have to.

Do you have any questions at this point?

I just want to confirm that you are happy for me to audio-record this interview for the purpose of my research.

**Start recording**

Can you start by telling me about which procedure you underwent and when?

Given this research is all about the patient experience, can you tell me how you feel overall about your experience?

Looking back, how do you feel about the information you received prior to your procedure?

What was your experience like with the staff in the Cath Lab? How did you find your interactions with both doctors and nurses?

Can you explain how you were feeling when you left the hospital following your procedure?

Did you feel prepared to leave the hospital following the procedure? Did you still feel ‘prepared’ when you got home? Can you elaborate on this?

What was it like for you when you got home?

How did you feel about the communication with clinicians before, during and after your procedure?

What instructions were you given, if any, about your discharge? What were you told you should look out for?

How were you supported at home following your procedure?

Did you feel that you would have been able to manage any potential complications following the procedure? How so?

What instructions or advice were you given regarding any follow up after you were discharged?

Overall, were you satisfied with your care?

Do you feel like there was anything missing from your care, in relation to your procedure and discharge preparation?

From your experience, do you have suggestions as to how Cath Lab can better support patients undergoing a procedure in Cath Lab?

Is there anything else you’d like to add before we conclude the interview?

**Close interview**

Thank you so much for your time today. If I have any further questions are you happy to be contacted again?
